# Imaging Biomarkers or Biomarker Imaging?

**DOI:** 10.3390/ph7070765

**Published:** 2014-06-25

**Authors:** Markus Mitterhauser, Wolfgang Wadsak

**Affiliations:** Radiochemistry and Biomarker Development Unit, Department of Biomedical Imaging and Image-guided Therapy, Division of Nuclear Medicine, Medical University of Vienna, A-1090 Vienna, Austria; E-Mail: markus.mitterhauser@meduniwien.ac.at

**Keywords:** tracer, molecular probe, PET, radiopharmaceutical

## Abstract

Since biomarker imaging is traditionally understood as imaging of molecular probes, we highly recommend to avoid any confusion with the previously defined term “imaging biomarkers” and, therefore, only use “molecular probe imaging (MPI)” in that context. Molecular probes (MPs) comprise all kinds of molecules administered to an organism which inherently carry a signalling moiety. This review highlights the basic concepts and differences of molecular probe imaging using specific biomarkers. In particular, PET radiopharmaceuticals are discussed in more detail. Specific radiochemical and radiopharmacological aspects as well as some legal issues are presented.

## 1. Introduction

The terms “imaging biomarker” and biomarker imaging” are currently used in a variety of different contexts. Therefore, a clear definition of these terms is pivotal. “Biomarker” has already been defined by the National Institutes of Health (NIH, Bethesda, MD, USA) director’s initiative on biomarkers and surrogate endpoints to be “a characteristic, that is objectively measured and evaluated as an indicator of normal biological processes, pathogenic processes, or pharmacologic responses to a therapeutic intervention” [1]. But this definition still gives room for a wide range of interpretations. On the one hand, it is evident that a biomarker has to be objectively measurable; on the other hand, it provides no information regarding the very nature of this measurable characteristic. From the given definition, a biomarker can be nearly every value associated with any process in any organism, even such trivial things as blood pressure, urinary pH or visual faculty. Going a bit further, a biomarker can be a molecular probe such as the calcium-score or levels of prostate specific antigen (PSA), or a functional indicator like extent of myocardial perfusion or renal clearance. Consequently, imaging biomarkers are all those biomarkers, which are determined by imaging techniques. Since there is no exclusivity to that term, examples are found in all methods: e.g., Nuchal translucency screening (ultrasound), sizing of adrenocortical incidentalomas (X-ray), BOLD-signal (magnetic resonance imaging—MRI), renal clearance (single photon emission computed tomography—SPECT).

Moreover, “imaging” is also a term with widespread use demanding for closer characterisation; coming from the Latin word *imago* meaning “picture”. Hence, imaging is a tool enabling the operator to obtain a picture of the organism. It can enable the assessment of the biological basis of the body, including changes due to disease, response to therapeutic intervention. Nowadays, imaging is mostly used as a synonym for visualisation (derived from the Latin word *video* meaning “to see”), although visualising processes and conditions are only part of imaging these things. The aspect that distinguishes imaging from mere visualisation is quantitation. This brings us back to the definition of biomarker being a measurable signal. For that reason, a visualisation biomarker could not exist! Imaging (and therefore imaging biomarkers) can be characterised as structural, functional and molecular, respectively. Hence, one should distinguish truly molecular information from rather functional or even structural measurements and restrain from thoughtlessly terming everything “molecular imaging”. So what makes imaging become molecular? The answer is simple: the visualisation and quantification of a molecular interaction between a molecular probe and a molecular target.

Recently, Lucignani strengthened the term “imaging biomarkers” and pointed out that they allow identifying measurable variables that are otherwise undetectable [2]. Furthermore, he stated that “moving the definition of an imaging biomarker from that of a technical probe to that of a biological variable” results in the recognition “that the use of imaging biomarkers transforms the role of molecular and anatomic imaging from technical to a fully clinical”. Bringing this to a synopsis, an imaging biomarker is “not a tool or a method but a measureable variable”.

Now, that we have defined imaging biomarkers can we simply rearrange these terms and consequently derive a definition of biomarker imaging as well? Unfortunately, it is not that simple and thus requires further reflection. Since biomarker imaging traditionally is understood as imaging of molecular probes, we highly recommend to avoid any confusion with the previously defined term “imaging biomarkers” and, therefore, only use “molecular probe imaging (MPI)” in that context. Molecular probes (MPs) comprise all kinds of molecules administered to an organism which inherently carry a signalling moiety. This moiety can for example be a radionuclide, a fluorescent chromophore, a paramagnetic core or a gas-filled or gas-producing nanostructure. The resulting signal has to be visualised and quantified by dedicated imaging procedures and technologies [3,4,5,6,7,8]. Hence, MPI always leads to an imaging biomarker; but imaging biomarkers do not necessarily require MPI! However, imaging biomarkers require a molecular imaging modality/technique in order to measure them (e.g., magnetic resonance spectroscopy doesn’t use molecular probes but can detect imaging biomarkers and is considered a molecular imaging technique).

## 2. PET & MRI: Selected Examples

The blood-oxygen-level dependent (BOLD) signal is an indirect measure for the local oxygenation level in tissue [9]. It is quantifiable and derives from the application of MR imaging. Therefore, it follows the definition of an IB. But it’s definitely not MPI: no MP is administered to achieve the resulting information.

Inorganic gadolinium (Gd) compounds as contrast enhancers for MRI are MPs [10]. Depending on physiological or pathophysiological status or changes in perfusion, these MPs can be quantified using MR technology. Thus, the imaging biomarker in that case is the extent of perfusion of the tissue measured through the Gd signal.

[^18^F]FMISO ([^18^F]fluoromisonidazole) is an example of an MP used in positron emission tomography (PET) for the assessment of hypoxia [11,12]. It is taken up by tissue and, in hypoxic cells, subsequently chemically changed and trapped. In this case, the biomarker is the extent of hypoxia in the investigated tissue indirectly measured through the uptake of [^18^F]FMISO. Comparing these complementary techniques to assess information on oxygen levels, it is evident that the quality and depth of the information is different.

[^18^F]FDG (2-deoxy-2-[^18^F]fluoro-d-glucose) is a radiolabelled analogue of glucose. Like its parent compound it is transported through the blood stream and taken up by cells through specific glucose transporters (GLUT). GLUT is expressed by cells in correlation to its energy demand. Therefore, tumour cells, inflammatory tissue or myocytes as well as neurons exhibit up-regulated GLUT. After being taken up by the cell, the fate of [^18^F]FDG and native glucose is different: due to the substitution of a hydroxyl moiety by the ^18^F-label in the molecule the biochemical catabolism is altered. In the first step of glycolysis, both glucose and [^18^F]FDG are phosphorylated by hexokinase enzyme. Subsequently, the glucose molecule is rearranged by an isomerase enzyme which is not able to use [^18^F]FDG as substrate. The ^18^F-substituent is hindering this transformation. Since hexokinase activity is the “driving force” in the cell to gain energy out of sugar molecules, it is—more or less—unidirectional allowing for almost no back-reaction. Hence, [^18^F]FDG is trapped within the cell and its uptake correlates to the energy demand of the tissue [13,14,15]. In this case, the MP is [^18^F]FDG; and the IB is a combination of hexokinase activity and GLUT expression, both representing a surrogate for energy demand.

^68^Ga-DOTANOC (*i.e.*, ^68^Ga-labelled l-cysteinamide, *N*-[[4,7,10-tris(carboxymethyl)-1,4,7,10-tetraazacyclododec-1-yl]acetyl]-d-phenylalanyl-l-cysteinyl-3-(1-naphthalenyl)-l-alanyl-d-tryptophyl-l-lysyl-l-threonyl-*N*-[(1*R*,2*R*)-2-hydroxy-1-(hydroxymethyl)propyl]-,cyclic(2-7)-disulfide), a radiolabelled peptide from the octreotide family, selectively targets somatostatin receptors [16,17]. In contrast to the endoligand, somatostatin (15 amino acids), ^68^Ga-DOTANOC represents only the active binding sequence of 8 amino acids connected to a DOTA chelator which enables the complexation of radiometals such as ^68^Ga. In contrast to “traditional” PET radionuclides (*i.e.*, ^18^F, ^11^C, ^13^N,) that are produced in a cyclotron, ^68^Ga is produced in a ^68^Ge/^68^Ga-radionuclide generator [18]. The somatostatin receptors are over-expressed in a variety of neuroendocrine tumours (NETs) and, therefore, represent an interesting and important target for clinical diagnosis and treatment [19,20]. Binding of ^68^Ga-DOTANOC to these receptors enables visualisation and quantification of their extent of expression. Since the expression rate of somatostatin receptors can help to assess the dignity of the tissue and peripheral tissue involvement (staging) it represents a prospective surrogate marker for clinical outcome in many cases. Thus, in this case ^68^Ga-DOTANOC is the MP; and the IB is the somatostatin receptor expression rate.

From these selected examples, it is obvious that MPs allow for more streamlined information. Since this information derives from a distinct interaction of the MP with a molecular structure or mechanism it represents—and should therefore be called—“molecular imaging”. Of course, molecular imaging is not restricted to the field of nuclear medicine methods. Several new approaches (e.g., multi-modal nanoparticles, optical nano-tubes, functionalizes micro-bubbles) also fulfil the definition of a molecular signal; and of course, fluorescent, bioluminescent, ultrasound-, photoacoustic-, and MRI-compatible molecular imaging probes are also available.

Currently, there is great enthusiasm for the simultaneous acquisition of different modalities [21]. The combination of PET and CT entered clinical routine some years back [22] and is nowadays a common state-of-the-art technique. New PET scanners are not available without a CT any more. Lately, PET/MRI hybrid technology was pushed both by researchers and companies and has entered the commercial market within the last 2 years. Especially, the combination of PET and MRI techniques is very promising since the different types of quantitative information or contrasts from MR Imaging and spectroscopy, respectively [23], can be fused with those deriving from the tracer specific PET signal. Bringing these IBs together may lead to a better description—or even understanding—of (patho-)physiological processes on a molecular level. This could be the basis for true prospective and personalized diagnostics.

## 3. The Role of Radiopharmaceuticals in PET and PET/MRI

**Figure 1:**
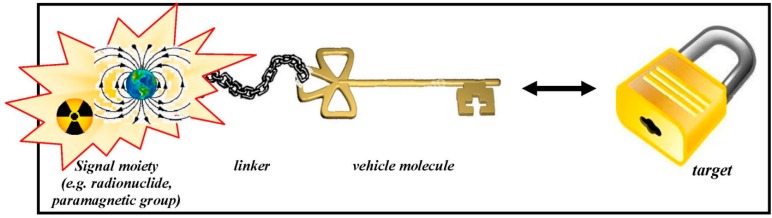
Schematic design of a molecular probe and its interaction with the target site.

Essentially, molecular probes consist of two functional entities: (1) a chemical backbone which is responsible for the *in vivo* interactions within the organism and (2) a signalling functionality which enables the accurate detection from outside of the organism with dedicated instrumentation that subsequently allows the visualisation and quantification for MPI [24]. The signalling moiety can be a radionuclide, a paramagnetic functionality or any other group that evinces a detectable and quantifiable signal. For a connection between the signalling core and the vehicle part of the molecule, a linker may be necessary for (chemical) stabilisation. An illustrative scheme is presented in Figure 1.

A radiopharmaceutical is a special MP bearing a radionuclide as signalling moiety. In the case of PET, the radionuclide has to belong to the class of positron emitters – examples are given in Table 1.

**Table 1:** Important PET radionuclides and their common way of production.

Nuclide	Production	Half-Life
^18^F (F^−^)	^18^O(p,n)^18^F	109.7 min
^18^F (F_2_)	^20^Ne(d,α)^18^F	109.7 min
^11^C	^14^N(p,α)^11^C	20.4 min
^13^N	^16^O(p,α)^13^N	10.0 min
^15^O	^14^N(d,n)^15^O	2.0 min
^64^Cu	^64^Ni(p,n)^64^Cu	12.7 h
^86^Y	^86^Sr(p,n)^86^Y	14.7 h
^76^Br	^76^Se(p,n)^76^Br	16.0 h
^68^Ga	^68^Ge/^68^Ga generator	67.6 min
^82^Rb	^82^Sr/^82^Rb-Generator	1.3 min
^124^I	^124^Te(p,n)^124^I	4.2 days

The history of radiopharmaceuticals is rather short, since radioactivity was not detected before the end of the 19th century. The first nuclear medical examinations of the thyroid were conducted in the 1940s and in 1958 the first clinical application of a positron emitting nuclide (*i.e.*, O-15) was described [25]. But only after considerable improvements in the instrumentation were implemented, PET became of interest as a diagnostic technique for the community. Ido *et al.* presented the first synthesis of FDG in 1978 [26] but only the significant improvements made by Hamacher and Coenen at the research centre Juelich (Germany) [27] led to the unrivalled success of this compound. Even today, FDG is the by far most important PET radiopharmaceutical accounting for approximately 90% of all clinical PET examinations worldwide [28]. In recent years, more specific and selective tracers have been developed, labelled with different PET nuclides, providing further insight in oncological, cardiac and neurological relationships. Radiopharmaceuticals are by definition radiolabelled drugs for *in vivo* application. They have been referred to as drugs as early as in 1960 [29].

The most widely used PET-nuclides are fluorine-18, gallium-68 and carbon-11. Since gallium-68 can be produced through a radionuclide generator its application can be implemented in radio-pharmacies without direct access to an on-site cyclotron facility. A radionuclide generator requires only limited shielding and allows preparing ^68^Ga-labelled radiopharmaceuticals directly in small facilities.

Due to its rather longer half-life, fluorine-18 can also be used in remote PET centres without their own cyclotron unit after shipping (satellite principle). In this manner, both ready-to-use ^18^F-labelled radiopharmaceuticals as well as the radionuclide solution itself for subsequent radiosyntheses can be obtained. Therefore, the use of ^18^F-labelled PET tracers is not strictly limited to facilities with an on-site cyclotron.

In contrast to that, Carbon-11 can only be used directly on site since its short half-life (20 min) does not allow any shipping to remote PET imaging units even if they are close by. This restriction derives from radiochemical yields and specific radioactivities which both decrease with time. Nevertheless, Carbon-11 is of great importance because it enables the preparation of unchanged, so called authentic, molecular probes.

Until now, the clinical application of other PET nuclides is limited to research and scientific purposes and has not yet found its way into broad clinical routine. This fact can be explained either by their short half-lives (^82^Rb, ^13^N, ^15^O) or their complex way of production (^124^I, ^76^Br, ^64^Cu, ^86^Y). Nevertheless, some of these nuclides will gain importance in the future since they have an isotope with suitable characteristics for therapeutic use (“theranostics”; e.g., ^124^I–^131^I, ^86^Y–^90^Y, ^64^Cu–^67^Cu) [30]. These theranostics enable the direct translation of a prospective diagnostic signal into a therapeutic application. It may even become possible to discriminate prospectively patients responding to nuclear medical treatment (curative and palliative) from non-responders. This concept is a clear example for personalized (nuclear) medicine.

Since radioactive molecular probes are chemically indistinguishable from their stable (*i.e.*, non-radioactive) parent compounds the organism is not able to differentiate between these two forms of the same compound. But this is only true for the exact identical substances—one with a radionuclide and the other with a non-radioactive isotope (belonging to the same element); e.g., [^18^F]FDG *vs.* ^19^F-FDG, [^11^C]methionine *vs.* ^12^C-methionine. It should be of note that [^18^F]FDG and glucose are not strictly the same chemical compound and therefore a distinct difference in their biochemical behaviour is observed! 

**Figure 2:**
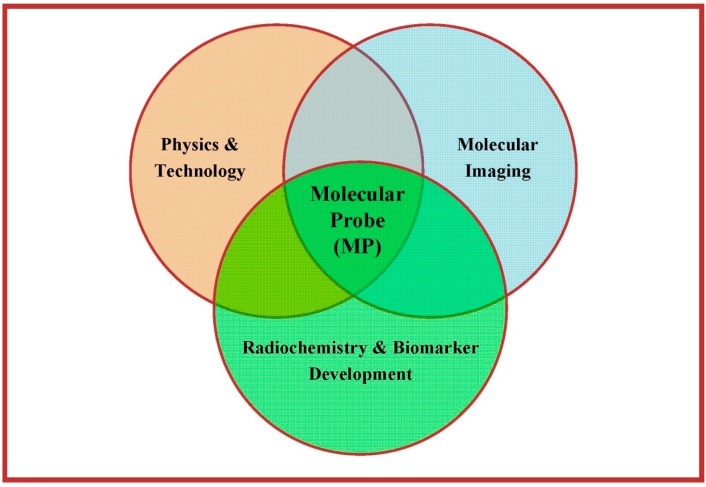
Necessary interplay of the three major disciplines involved in successful application of PET/MRI—the central role of the molecular probe is highlighted.

Essentially, three major disciplines have to interact and cooperate closely to enable a successful application of MPs in PET, PET/CT and PET/MRI, respectively, to gain IBs in a clinical setting: (medical) physics and technology, radiopharmaceutical sciences (radiochemistry and biomarker development) and (clinical) molecular imaging (see Figure 2). All experts and scientists involved in the whole process—regardless of their profession and provenience—have to invest in understanding of the basic principles and comprehension of the different scientific languages used by the different disciplines. Hence, understanding of radiopharmaceutical issues is as important as understanding of medical physics or molecular imaging as such. Overall, the molecular imaging probe (e.g., the PET tracer) plays THE central role in the whole process because it determines the achievable information and, hence, the subsequent processing through modelling techniques and its scientific and clinical interpretation. On the one hand, signal detection, resolution and computational processing are based—although not exclusively—on the chosen radionuclide with its particular physical characteristics: the selection of the PET radionuclide should focus on the following presumptions:

- Availability of the radionuclide;
- Physical characteristics of the radionuclide;
- Radiochemical issues; and
- Radiopharmacological issues.

On the other hand, it is pivotal to comprehend the (bio-)chemical properties of the molecular vehicle, especially for molecular modelling and dosimetry. Of particular interest is the deep understanding of:

- Binding and/or uptake characteristics in terms of affinity, selectivity and unspecific binding;
- Pharmacokinetics yielding information on input function, elimination rate and principal fate of the compound;
- Metabolic issues with respect to formation of metabolites potentially interfering with the original signal and stability considerations;
- Involvement in different (patho-)physiological processes such as parallel representation of both vital tumour tissue and inflammation or combined information on perfusion and hypoxia; and
- Pharmaceutical issues referring to interactions of molecular probes and co-medication as well as influence of the pharmaceutical formulation of the molecular probe (pharmacokinetic and pharmacodynamic considerations; *nota bene*: pharmacodynamic effects are in general of no interest in molecular imaging using radiopharmaceuticals because of the minuscule mass of compounds that is administered. Therefore, pharmacodynamics are not considered herein).

Even a basic understanding of radiochemical issues and preparation techniques is required for the application of PET/MRI in a scientific or clinical setting (e.g., formation of potential side-products, interaction patterns of both labelled and unlabelled compounds with the target structure, specific radioactivity and its time dependence).

The molecular probe—and especially the targeting component—has to display a high degree of specificity and selectivity towards the target site. Important targets are:

- Receptor systems and their subtypes;
- Transporter proteins;
- Antigens;
- Specific intra- or extracellular enzymes;
- Alterations in gene and protein expression;
- Changes in physiology and metabolism, such as hypoxia or differences in vascularisation and perfusion;
- Energy turnover.

The molecular probes either interact directly with the aforementioned targets and processes or enable an indirect assessment of imaging biomarkers.

In a pathophysiological state, these targets and/or processes may be altered distinctively in comparison to their baseline status. Hence, the quantifiable signal resulting from the interaction of the MP with these molecular targets may also be changed considerably. For example, in many tumours the expression rate of some receptors or transporters as well as enzymatic activity and representation of antigens on the cell surface is modified and these changes may serve as a suitable predictor for stage and allocation of these tumours (IB).

## 4. Specific Radiochemical Considerations

Radiopharmaceutical chemistry focuses on activation of the radionuclide, radiolabelling procedures and purification and formulation of the product. Since the chemical form of the radionuclide (coming from the cyclotron or a generator system) is normally not predisposed for direct labelling reactions, the first step in the labelling sequence is an activation reaction. For instance, [^18^F]fluoride delivered from the target is received as an aqueous solution that is chemically inactivated due to its shell of water molecules and therefore has to be removed from its aqueous environment. This is achieved by azeotropic drying with acetonitrile in presence of an aminopolyether as phase transfer catalyst. The next step is a coupling reaction directly with or incorporation into the predesigned precursor compound. This substitution reaction generally demands elevated temperature or other ways of chemical activation (e.g., microwave assistance [31,32], microfluidic conversion [33,34], high pressures). Due to the very nature of these micro- or even nano-scale reactions, dedicated equipment is required. In contrast to traditional chemistry, it is usual to waive relative yields in favour of reduction of reaction time [24]. As a matter of fact, due to the short-lived nature of the commonly used PET-radionuclides (*cf.* Table 1) increased relative reaction yields are often outbalanced by the physical decay during longer reaction durations. Thus, the absolute radiochemical yields (at the end of synthesis (EOS); measured in GBq) are usually higher when using short reaction times (*i.e.*, 1–20 min). This means that, in contrast to conventional organic chemistry, the main aim is not a quantitative conversion but the maximum achievable total yield of radioactivity (in GBq). It is therefore sometimes the better choice to sacrifice chemical conversion output (in %). This is of particular interest for Carbon-11: if for example in a ^11^C-methylation reaction the conversion yield is 15% after 1 min at room temperature (which is ridiculously low and an unacceptable value for an organic chemist) and increases to 50% after 35 min under heating at 80 °C (which means that for heating up and cooling down an other 5 min are required) the total achievable yield is lower for the latter conditions! Other differences to conventional chemistry are:

- There is no stoichiometry between the reaction partners (*i.e.*, the radionuclide and the precursor molecule)! A huge excess of the precursor is present in the reaction solution compared to the amount of radionuclide. An illustrative extrapolation reflecting this imbalance would be to react a whole shipload of precursor material with as little as one single sugar cube of radionuclide!
- Reactions that are unsuccessful under normal chemical circumstances are sometimes successful in radiochemistry and *vice versa*.
- Due to the very low mass of reaction partners (often 1 mg precursor or less), the use of special miniature equipment is demanded.
- Working with high amounts of radioactivity (several tens of GBq) requires powerful radiation protection devices. All radiolabelling procedures have to take place in a fully lead shielded environment (dedicated hot cells) under remote control using special equipment for manipulation like tele-tongs or robotic arms.
- For the routine delivery of radiopharmaceuticals with maximum quality, the radiosyntheses are performed in automated synthesis modules which can be remotely controlled from outside the shielded hot cells (quality assurance). The production sequences are programmed in advance based on optimization procedures and performed automatically with constant and reproducible quality.
- Any carrier (stable isotope of the radionuclide taking part in the reactions) influences the radiochemical conversion leading to a significant amount of indistinguishable, non-radioactive product that “dilutes” the final radiopharmaceutical. This is followed by a significant change in stoichiometry. This carrier also determines the maximum achievable specific radioactivity which is a measure for the radioactivity per mass unit (e.g., GBq/µmol or MBq/nmol) [35,36]. Such carrier is introduced into the radiosynthetic procedure either on purpose (due to production obligations) or by unwanted “contamination” of reaction partners or solvents. [36,37] In case of Carbon-11, even the ^12^C-CO_2_ always present in air may contribute significantly to a reduction in specific activity. In case of fluorine-18, the isotopically enriched target material (water, >98% ^18^O) also contains traces of non-radioactive fluorine-19 as dissolved fluoride anions. Typically, the content of ^19^F-fluoride is specified as <0.1 mg/L (=100 ppb) [38,39,40] and the total target volume usually is 2–5 mL. Hence, up to 0.2–0.5 ng of ^19^F-fluoride are present—that equals 10–26 pmol. However, 100 GBq of [^18^F]fluoride equal only 30.0 pg (=1.6 pmol) Therefore, up to a 16-fold excess of non-radioactive fluoride may be present in terms of amount of substance!
- When working with solutions with high radioactivity concentrations, one has to consider radiolysis as a major factor reducing product purity leading to formation of unwanted by-products. Especially when dealing with batches that are to be shipped (e.g., [^18^F]FDG and [^18^F]fluoride for labelling) concentrations up to 35 GBq/mL (700 GBq in 20 mL) can be reached [41]! In these cases, addition of stabilizers to the product matrix may be indicated.

Radiolysis may also occur during the production process (also for carbon-11), particularly when applying excessive heating and evaporation to dryness *under vacuo* for product purification [42]. Hence, alternative methods under mild conditions are usually applied in radiopharmaceutical chemistry, such as heating under microwave conditions or reduction of residual solvents by solid phase extraction (SPE) techniques [43]. Overall, high starting activities (leading to satisfactory final activities) and measures to suppress radiolysis have to be thoughtfully balanced.

## 5. Specific Radiopharmacological Considerations

With respect to the short-lived nature of the used radionuclides, also some distinct differences in the (radio-)pharmacological behaviour of the MPs occur that have to be taken into consideration when dealing with radiopharmaceuticals.

**Figure 3:**
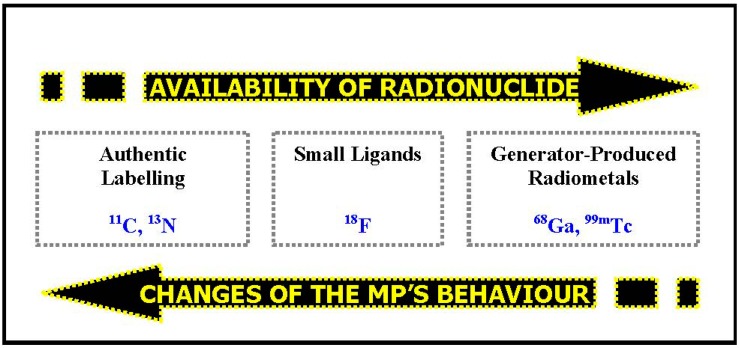
The radiochemist’s dilemma: availability of radionuclides *vs.* potential changes of *in vivo* behaviour. Usually, the better the availability of the radionuclide (e.g., Tc-99m > F-18 > C-11) the more pronounced the observed changes of the MP’s properties regarding e.g., uptake, binding characteristics and pharmacokinetics.

- As already mentioned earlier in this review, the specific radioactivity is an essential parameter for the quantification and visualisation of the MP’s signal—the imaging biomarker. In many (patho-)physiological imaging procedures the actual number of target sites is limited and the process of binding between the MP and the specific target (e.g., receptor subtype, transporter, antigen binding pocket) is saturable. Then, the maximum available binding sites (B_max_) determine the maximum achievable signal. Specific radioactivity (as the measure for the ratio of non-radioactive to radioactive molecules with the identical structure and binding characteristics) therefore allows predicting the amount of detectable signal, reflecting the ratio of signal-evincing to signal-erasing binding. Consequently, the required specific radioactivity has to be higher when the density of the target sites is lower. *Nota bene*, the specific radioactivity should be kept constant throughout a (quantified) clinical study and should always be included as a co-variable in statistical analysis of the data (e.g., binding potential).
- The radionuclide as the signalling function of the MP only allows for the allocation and quantification of the annihilation events. But it does not reflect the integrity of the MP. Thus, it might occur that the radionuclide (or a small part of the molecule which includes the signalling radionuclide) is cleaved from the intact parent molecule through active metabolism. Subsequently, the measured signal would be wrongly assigned and quantification would be systematically biased. Therefore, knowledge on and quantification of the metabolic stability of the MP (both in target tissue and blood stream) are pivotal.
- The availability of the desired radionuclide and the potential alterations in the *in vivo* behaviour of the MP due to the insertion of this radionuclide unfortunately point into opposite directions. (*cf.* Figure 3) For example, gallium-68 is readily available through a radionuclide-generator system but, since it is a radiometal, has to be integrated into the MP’s backbone by addition of a bulky chelating group and complexation. This leads to a drastic change in the molecular structure and therefore the MP’s *in vivo* behaviour becomes unpredictable. This is true not only for gallium-68 but for all radiometals and leads to probable changes in pharmacokinetics of the so-labelled radiopharmaceuticals. As a consequence, there is—despite significant efforts throughout many years—only one MP labelled with a radiometal targeting a receptor system in the brain that found its way into clinical application, namely ^99m^Tc-TRODAT [44,45]. Moreover, interactions between radiometal and chelator within a complex are usually much weaker than covalent binding. On the other hand, carbon-11 labelled radiotracers represent the authentic (unchanged) molecule. carbon-11 is always bound covalently within the vehicle molecule, most often as a [^11^C]methyl group attached to an amine, hydroxyl or carboxyl moiety. No isotopic effect can be observed and, consequently, organisms are not able to distinguish between the original compound and its ^11^C-labelled analogue. Finally, fluorine-18 is also attached covalently to the parent molecule. Since normally the target molecules do not bear a fluorine atom in their chemical structure the label has to be added (and not substituted). Although the structural change may appear small it can not be excluded that this will lead to pronounced changes in the MP’s characteristics—both, *in vitro* and *in vivo*. This could be in both directions: it might lead to reduced affinity, stability or selectivity but could also lead to ameliorated *in vivo* behaviour [24].

The choice of the used radionuclide (and MP) will therefore always be a compromise between availability and economical considerations on the one hand, and physical properties, radiolabelling possibilities and radiopharmacological issues on the other hand.

## 6. Regulatory Aspects and Quality Assessment

By law in most countries, PET-radiopharmaceuticals are drugs. Hence, a manifold of regulatory aspects have to be addressed prior to release for human. Since the majority of radiopharmaceuticals are administered intravenously, these parenteral drugs have to follow even stricter regulations. A specific monograph in the European Pharmacopoiea has been established to generally cover radiopharmaceutical preparations [46]. Furthermore, there are more than 50 specific monographs for radiopharmaceuticals, with growing numbers with every new edition. These monographs provide details on preparation, precursors, and tests of identity, purity, chemical purity, radionuclidic purity, radiochemical purity, and measurement of radioactivity, residual solvents, sterility, endotoxines and even labelling of the containers. The short half-life especially of PET radionuclides allows for “parametric” release of the product under specific circumstances (=the final product may be released by a radiopharmacist before completion of the whole QC procedure). This implicates that the methods used for the preparation of the radiopharmaceutical have to be safe and robust and included in a general quality management system.

Finally, the time for multi-modal molecular imaging probes has finally come and the simultaneous gathering of molecular information on the basis of imaging biomarkers and hybrid systems has already started, paving its way into personalized medical diagnosis and treatment.

## References

[1] Biomarkers Definitions Working Group (2001). Biomarkers and surrogate endpoints: Preferred definitions and conceptual framework. Clin. Pharmacol. Ther..

[2] Lucignani G. (2001). Imaging biomarkers: From research to patient care—A shift in view. Eur. J. Nucl. Med. Mol. Imaging.

[3] Peng B.H., Levin C.S. (2010). Recent development in PET instrumentation. Curr. Pharm. Biotechnol..

[4] Mittra E., Quon A. (2009). Positron emission tomography/computed tomography: The current technology and applications. Radiol. Clin. N. Am..

[5] Lecomte R. (2009). Novel detector technology for clinical PET. Eur. J. Nucl. Med. Mol. Imaging.

[6] Lewellen T.K. (2008). Recent developments in PET detector technology. Phys. Med. Biol..

[7] Spanoudaki V.C., Ziegler S.I. (2008). PET & SPECT instrumentation. Handb. Exp. Pharmacol..

[8] Townsend D.W. (2008). Positron emission tomography/computed tomography. Semin. Nucl. Med..

[9] Forster B.B., MacKay A.L., Whittall K.P., Kiehl K.A., Smith A.M., Hare R.D., Liddle P.F. (1998). Functional magnetic resonance imaging: The basics of blood-oxygen-level dependent (BOLD) imaging. Can. Assoc. Radiol. J..

[10] Hermann P., Kotek J., Kubícek V., Lukes I. (2008). Gadolinium(III) complexes as MRI contrast agents: Ligand design and properties of the complexes. Dalton Trans..

[11] Lee S.T., Scott A.M. (2007). Hypoxia positron emission tomography imaging with 18f-fluoromisonidazole. Semin. Nucl. Med..

[12] Padhani A. (2006). PET imaging of tumour hypoxia. Cancer Imaging.

[13] Smith T.A. (1998). FDG uptake, tumour characteristics and response to therapy: A review. Nucl. Med. Commun..

[14] Abouzied M.M., Crawford E.S., Nabi H.A. (2005). 18F-FDG imaging: Pitfalls and artifacts. J. Nucl. Med. Technol..

[15] Pauwels E.K., Ribeiro M.J., Stoot J.H., McCready V.R., Bourguignon M., Mazière B. (1998). FDG accumulation and tumor biology. Nucl. Med. Biol..

[16] Breeman W.A., de Blois E., Sze Chan H., Konijnenberg M., Kwekkeboom D.J., Krenning E.P. (2011). (68)Ga-labeled DOTA-peptides and (68)Ga-labeled radiopharmaceuticals for positron emission tomography: Current status of research, clinical applications, and future perspectives. Semin. Nucl. Med..

[17] Lopci E., Nanni C., Rampin L., Rubello D., Fanti S. (2008). Clinical applications of 68Ga-DOTANOC in neuroendocrine tumours. Minerva Endocrinol..

[18] Prata M.I. (2012). Gallium-68: A new trend in PET radiopharmacy. Curr. Radiopharm..

[19] Reubi J.C., Laissue J., Waser B., Horisberger U., Schaer J.C. (1994). Expression of somatostatin receptors in normal, inflamed, and neoplastic human gastrointestinal tissues. Ann. N. Y. Acad. Sci..

[20] Reubi J.C., Krenning E., Lamberts S.W., Kvols L. (1990). Somatostatin receptors in malignant tissues. J. Steroid Biochem. Mol. Biol..

[21] Zaidi H., Montandon M.L., Alavi A. (2010). The clinical role of fusion imaging using PET, CT, and MR imaging. Magn. Reson. Imaging Clin. N. Am..

[22] Beyer T., Townsend D.W., Brun T., Kinahan P.E., Charron M., Roddy R., Jerin J., Young J., Byars L., Nutt R. (2000). A combined PET/CT scanner for clinical oncology. J. Nucl. Med..

[23] Moser E., Stadlbauer A., Windischberger C., Quick H.H., Ladd M.E. (2009). Magnetic resonance imaging methodology. Eur. J. Nucl. Med. Mol. Imaging.

[24] Wadsak W., Mitterhauser M. (2010). Basics and principles of radiopharmaceuticals for PET/CT. Eur. J. Radiol..

[25] Ter-Pogossian M.M., Powers W.E. (1958). Radioisotopes in Scientific Research.

[26] Ido T., Wan C.N., Casella V., Fowler J.S., Wolf A.P., Reivich M., Kuhl D.E. (1978). Labeled 2-deoxy-d-glucose analogs. 18F-labeled 2-deoxy-2-fluoro-d-glucose, 2-deoxy-2-fluoro-d-mannose and ^14^C-2-deoxy-2-fluoro-d-glucose. J. Label. Compd. Radiopharm..

[27] Hamacher K., Coenen H.H., Stoecklin G. (1986). Efficient stereospecific synthesis of no-carrier-added 2-[^18^F]-fluoro-2-deoxy-d-glucose using aminopolyether supported nucleophilic substitution. J. Nucl. Med..

[28] Rowe C., Keefe G.O., Scott A.M., Tochon-Danguy H.T. (2005). Positron emission tomography in neuroscience. Medicamundi.

[29] Briner W.H. (1960). Radiopharmaceuticals are drugs. Mod. Hosp..

[30] Del Vecchio S., Zannetti A., Fonti R., Pace L., Salvatore M. (2007). Nuclear imaging in cancer theranostics. Q. J. Nucl. Med. Mol. Imaging.

[31] Kallmerten A.E., Alexander A., Wager K.M., Jones G.B. (2011). Microwave accelerated labeling methods in the synthesis of radioligands for positron emission tomography imaging. Curr. Radiopharm..

[32] Velikyan I., Beyer G.J., Långström B. (2004). Microwave-supported preparation of (68)Ga bioconjugates with high specific radioactivity. Bioconjug. Chem..

[33] Wang M.W., Lin W.Y., Liu K., Masterman-Smith M., Kwang-Fu Shen C. (2010). Microfluidics for positron emission tomography probe development. Mol. Imaging.

[34] Elizarov A.M. (2009). Microreactors for radiopharmaceutical synthesis. Lab Chip.

[35] Zeevaart J.R., Olsen S. (2009). Recent trends in the concept of specific activity: Impact on radiochemical and radiopharmaceutical producers. Appl. Radiat. Isot..

[36] De Goeij J.J.M., Bonardi M.L. (2005). How to define the concepts specific activity, radioactive concentration, carrier, carrier-free and no-carrier added. J. Radioanal. Nucl. Chem..

[37] Welch M.J., Redvanly C.S. (2002). Handbook of Radiopharmaceuticals: Radiochemistry and Applications.

[38] Rotem Industries Hyox 18 Enriched Water. http://www.rotem-medical.com/hyox18/.

[39] Huayi Isotopes Co. Oxygen 18 Water 95 atom %. Specifications. http://www.huayi-isotopes.com/EnProductShow.asp?ID=136.

[40] Isoflex Oxygen-18 Enriched Water—98 atom %. Product Specifications. http://www.isoflex.com/imaging/O-18_wt-98.html.

[41] Eberl S., Eriksson T., Svedberg O., Norling J., Henderson D., Lam P., Fulham M. (2012). High beam current operation of a PETtrace^TM^ cyclotron for 18F^−^ production. Appl. Radiat. Isot..

[42] Fukumura T., Nakao R., Yamaguchi M., Suzuki K. (2004). Stability of 11C-labeled PET radiopharmaceuticals. Appl. Radiat. Isot..

[43] Lemaire C., Plenevaux A., Aerts J., del Fiore G., Brihaye C., le Bars D., Comar D., Luxen A. (1999). Solid phase extraction—An alternative to the use of rotary evaporators for solvent removal in the rapid formulation of PET radiopharmaceuticals. J. Label. Compd. Radiopharm..

[44] Meegalla S.K., Plössl K., Kung M.-P., Chumpradit S., Stevenson D.A., Kushner S.A., McElgin W.T., Mozley P.D., Kung H.I.E. (1997). Synthesis and characterization of Tc-99m labeled tropanes as dopamine transporter imaging agents. J. Med. Chem..

[45] Kung H.F., Kim H.-J., Kung M.-R., Meegalla S.K., Plössl K., Lee H.-K. (1996). Imaging of dopamine transporters in humans with technetium-99m TRODAT-1. Eur. J. Nucl. Med..

[46] Radiopharmaceutical Preparations (2005). Radiopharmaceutica, 5.0/0125. European Pharmacopoeia (Europaeisches Arzneibuch).

